# A Brief Treatise on the Common Diseases of the Maxillary Sinus

**Published:** 1889-06

**Authors:** H. H. Schuhmann


### COMMUNICATIONS.

### A Brief Treatise on the Common Diseases of the Maxillary Sinus.

BY H. H. SCHUHMANN.

To form a correct idea of the various pathological conditions in the antrum of Highmore, it will be necessary to briefly recall to memory some of the most important and well known anatomical features of the region we intend to dwell upon.

The superior maxillary bone when closely examined is seen not to be as clumsy and heavy a mass as the first glance might lead us to suspect. It is composed of a number of processes so arranged as to enclose a large hollow, termed the antrum of Highmore, after the English anatomist Nathaniel Highmore, who first described minutely its pathological conditions. In examining different bones it will be seen that this cavity differs considerably in length, depth, and thickness of its parietes, in fact in almost every sense. To thoroughly familiarize one's self with the boundaries, etc., of this sinus, it would be necessary to examine these variations in a large number of bones. As all these points have very practical significations it is a subject that is well worthy of study.

The cavity is irregular, triangular shaped; its apex, which is directed outward, is formed by the malar process; its base by the outer nasal wall; its floor by the alveolar process; its anterior wall by the facial, and its posterior by the zygomatic surface. The walls of the antrum are generally extremely thin, especially may this be said of the orbital plate, also immediately above the canine fossa, upon the buccal wall, and above and between the palatal roots of the upper molars. A knowledge of this will explain how it is possible that engorgement of this cavity by solid growths or liquid accumulations in it bulges some part or other of the enclosing parietes, easily breaking through at one or more places, or perhaps lifting the orbital plate so as to push the eyeball out of its cavity and laying it more or less on the cheek; or it may be distorting the hard palate or nose by the same forces.

The inner wall as already stated is formed by the outer wall of the nose; in the disarticulate skull this inner wall will not be found formed by the maxillary bone, but a large aperture will be seen in its stead. In the articulated bones this opening is closed by the palatine process of the ethmoid, the inferior turbinated and palate bones; only leaving two very small openings, one of which is usually found closed by a fold of the lining membrane of both nasal and antral cavity; the other opening, when the parts are in a healthy condition, is just about large enough to admit a fine probe. This opening connects the antrum with the middle meatus of the nose. Some operators have used this opening for the introduction of a tube as a convenient receptacle for injecting purposes, but as this is a rather difficult way of obtaining access more convenient modes have been suggested.

In many instances the maxillary sinus is found to be divided into two or more communicating compartments by bony spiculæ running across its floor (this feature must be taken into consideration when free drainage of this cavity is to be obtained) no such pockets are in such a case to be left, but as a rule these laminæ should be broken down with chisels or gouges. Quite often the floor will be found studded with small projections. When these very thin septæ are opened the apex of the roots of the teeth will appear. The roots of several of the upper teeth have a close relationship with the floor of the antrum and it is not uncommon to find especially the palatine roots of second molars penetrate the sinus, thus associating their membranes and furnishing a contiguity if not a continuity of structure.

On the posterior walls are the posterior dental canals, transmitting the posterior dental vessels and nerves to the teeth, this will show why in some antral diseases (especially accumulations of fluid or solid matter) the pain is so frequently felt in a number of teeth, or maybe in some particular molar which seems to be as sound as any other, and really may be, but its vessels and nerves are being infringed upon before they reach the organ they were destined for.

Very few writers of pathology and therapeutics have given this subject due consideration, though some antral troubles are of a very dangerous cast. Some are of a more fear-exciting type of disease than any the practitioner may be called upon to treat. Some diseases of the maxillary sinus may be of a very benign sort as long as they remain in the incipient state, but if left uncared for for any length of time they may become of malignant character and defy all skill and knowledge of any physician or surgeon and the disease only ends when the ufortunate sufferer does.

All of these diseases are not as dangerous especially when attended to in their incipient stage and will often submit to proper treatment quickly and maybe with but little discomfort to the patient. As in any part of the economy, so also here, is the form and gravity of illness greatly varied by the state of the general health and specific diathesis. We can very well understand that a slight irritation in one robust and healthy may start up a little inflammation, whilst the same cause in a weak and anæmic subject may produce a most horrible indolent ulcer and other destructive changes in the tissues. In some patients a morbid condition of the maxillary sinus may exist for a number of months, yes years, without ever being noticed, and in others again the same trouble may extend with such rapidity that even if timely treated it will not submit, but may, if only a slight inflammation, cause caries and breaking down of the surrounding osseous tissues, and may even end with death of the sufferer.

Diagnosis of diseases in the antrum is not always easy though some antral troubles can be clearly recognized without much difficulty. The cavity of the antrum is so large that percussion will often give important information as to whether the sinus is full or empty. The proper place to tap the sinus for diagnosing purposes, for introduction of a probe or trephine, is above the second bicuspid tooth, about one inch above the margin of the gum. It may safely be assumed that in a vast majority of cases of diseased condition of the antrum the danger to be apprehended arises more frequently from neglect than from any necessary fatal character of the malady.

In forming a prognosis the circumstances to be considered are the general health of the patient, his susceptibility to inflammation and fever, and the rapidity with which the disease spreads. It is to be remembered, as to solid growths, that malignant tumors, grow rapidly, are generally painful in themselves, cause more or less constitutional disturbances, and have a tendency to degenerate. If the patient's health be in good condition, the surrounding hard and soft structures in a perfect state, the functional operations in the economy unaffected and the patient is not very old or feeble, the prognosis will be a favorable one, but if the bones of the face and nose are implicated in the disease and the general constitution run down, all medical or surgical aid will be of little or no avail.

In studying in almost any medical book you will find that the general constitutional health stands in direct proportion with the malignancy of disease; then any treatment for disease of the antrum will comprise generally more or less constitutional treatment; our skill and knowledge should be utilized for the general welfare of our patients, as well as being applied locally.

The antral affections are all more or less loathsome and give sufferers great inconvenience, the discharge in some of these diseases being very fetid and secreted through the nose or some opening made by the surgeon, or through a fistula, are so extremely unpleasant as to continually cause nausea of the patient and also being very disagreeable to the attendant of the sufferer. As already stated, generally antral diseases are of slow and increasing vigor, and may be present weeks or even months being unnoticed, at least not very much, generally being confounded with some slight oral or nasal trouble, which the physician or surgeon thinks unimportant until graver symptoms put in their appearance.

Most diseases of the antrum are due directly to dental complications. There are many connected with this sinus, though as different varieties of tumors, which are as likely to occur here as in any other situation on mucus membranes, dental irritation may be a predisposing cause for many of these. The general sources of the morbid conditions in the antrum may be divided into two classes. First, by roots of teeth reaching into it, and secondly, from the lining membrane being affected likewise throughout mucous linings; troubles derived from the latter cause do not vary essentially from other morbid mucoid affections, except as modifications are made by the situation. Professor Garretson thinks that syphilis does not often attack the antrum of Highmore very readily. In the secondary stage the disease may first show itself in an antral trouble, he says, but practice shows that this is rarely the case. In the tertiary stage the disease very rarely attacks the maxillary sinus unless from continuity of structure. Through continuity by being so closely related to the oral cavity through the hard palate (a common seat of syphilitic destructions) and toward the nose by the turbinated bones, he is of the opinion that the mercurial effects on the periodental membrane is more frequently the source of offense, rather than the specific condition. Mucous membranes are very prone to attacks of the venereal deterioration, and I think that syphilis does attack the antrum for that reason, although the malady may be greatly increased, and also predisposed, in character by mercurial diathesis. Poorly performed dental operations are sometimes indirect causes of diseases in the antrum; root fragments may be left in their alveoli (or in the antrum if they have penetrated its floor) after attempts at extraction. Such debris will act as foreign bodies and produce inflammation and may go on to further destruction and suppuration.

As already stated most diseases in this region are due to dental disturbances and as the first and maybe most common one, let us consider a disease, which was wrongly termed Abscess of the Antrum, it is a misnomer. It is not a suppuration of the inflamed parenchyma, but is simply an occlusion of a purulent secretion in a cavity, the excretions coming from the lining mucous membrane. The relation between the maxillary sinus and the roots of some of the superior teeth varies greatly both as regards the degree in which some roots approach or pierce the antral floor, and as to the number of roots over which the floor extends. A. W. Otto has written quite a good deal about the variation in size of the antrum and its relative position. Another circumstance in the anatomy of the antrum bearing markedly on what is termed "Abscess of the Antrum" is the variation in size of the antro-nasal opening. The lining membrane of the sinus is liable as all mucous membranes and especially the Schriderian membrane, of which this is a continuation, to inflammation and alteration in its secretions, both in regard to amount and kind. The sinus being impregnated with, and the mucus replaced by pus, accumulated in quantity. This latter assertion speaks for itself of another pathological condition, that is, that the absorptive power of the lining membrane decreases, when the diseases are such. The circumstances causing the altered and more abundant secretion depends on the fact that it may become occluded in the cavity, if the antro-nasal opening should close through slight swelling. Prof. Holmes describes this condition as an inflammatory effusion in an expending sac, which in many respects is quite similar to a deep seated abscess, though by no means identical with the pathological history or absolute anatomy. Most authorities agree that the cause for this affection may be enumerated in very close limits and are almost always due to the alveolar abscess in one of its stages or to inflammation of the tooth pulp (Holmes) extended to the mucous lining, either by continuity or by contiguity; most always then has its origin in some dental effection.

The symptoms vary, usually commencing with a feeling of fullness then a dull aching, throbbing, pulsating pain in the cheek with the other symptoms of inflammation as heat, redness and a fullness of the soft parts externally. Many authors now say that there is a purulent discharge from the corresponding nostril; there may be such a discharge, but that must not be relied upon, as the opening from the antrum to the middle meatus may have closed from the very beginning of the trouble, in fact may have been the very first pathological change. If the opening be not closed, then the patient will notice the superabundant fetid discharge, from the nostril, most in the evening, when he goes to bed and lets his face rest on the healthy side, as then the fluid accumulated during the day will run into the nose, or if he lies on the affected side, he will notice it when he arises. As long as this secretion remains purulent and watery, the disease is dropsy or antracele, but becomes abscess as soon as the fluid turns thick and degenerates. As the disease goes on and the pressure within the cavity augments by an increasing accumulation of fluid, the pain assumes a more throbbing and dissential character and is very severe; a little later, the same constitutional symptoms arise, which are generally present in alveolar abscess, the patient has chills and fever. A change in the local symptoms arises as the pressure within the cavity becomes greater, they manifest themselves more and more. Sometimes the features become so greatly distorted, as to render the patient unrecognizable. The whole jaw expands, the malar bone is pressed upwards; instead of a fossa beneath it, there is now a fullness and prominence; the molars of the affected side feel elongated, there is acute pericementitis, the elongation may partially be due to actual pressure from within the antrum, the concavity of the hard palate becomes either flattened or even convex, the nostril of that side is more or less closed, the floor of the orbit is raised and the eyeball considerably displaced. Vision may be so impaired with an antral abscess as to become permanently de fective, permanent amaurosis. Total blindness may result, though rarely by causing extreme anæmia of the optic nerve. Prof. Holmes says that the inflammation, accompanying abscess of the antrum is occasionally so severe as to implicate the periosteum, not only to the destruction of some parts of the maxilla, but extending beyond the structures, so as to involve the optic and other nerves in their passage from the cranium to their destination in the orbit; producing blindness and fixedness of the pupil on the affected side; these cases are rare, but several of them are on record; one occurred in the practice of Prof. Holmes at Guy's Hospital, under Mr. Pallack and another under Bruck.

The diagnosis of this affection is generally not very difficult. Besides to the signs and symptoms of the trouble, resort may be taken to fluctuation which can be felt. The pressure within has partially atrophied the walls of the cavity and they have become quite thin. They now convey a peculiar sensation to the touch, like the handling of dry parchment. To fully insure a correct diagnosis, and that is our aim at last, and sure method of testing the contents of the sinus is exploration.

A minute trocar and canula, not above half the size of a wheaten straw, is the kind Dr. Holmes makes use of, may be employed; if the contents be fluid a sufficient quantity to ascertain its nature. Frequently an ordinary abscess-needle is used instead of the trocar, but is not as reliable, as the fluid contents may be so thickened that they will not run in those narrow canals, such as are on abscess needles.

The ultimate results of these cases vary greatly occasionally, the abscess bursts into the nose, or it is often seen through the cheek, leaving considerable deformity by glueing the cheek to the bone and producing a disfiguring permanent scar unless later removed by a plastic operation. Occasionally the pus finds its way into the orbit, causing great displacement of the eyeball, temporary or even permanent blindness, and is finally evacuated through a fistulous opening at the inner or outer canthus.

The treatment for this trouble is generally not a very complicated one, though it may become so, if the disease is left to run its own course for any considerable length of time. If proper attention is paid to two points only, a cure can generally be effected without very great trouble, but no matter how extensive a treatment the sufferer may undergo, if these two points are not properly cared for, a cure will in no case be the result. The two points I have laid so very great stress upon, were taught by Prof. Martin, and are the two pillars upholding the treatment of any abscess; they are first *free drainage* and secondly *cleanliness*. Now, the next question is, how can this be attained? In many cases, general anæsthesia will not be necessary, but if the antrum is found, after exploration, to be divided into a number of small cavities, or if access to it must be obtained through an external opening through the cheek, then it may be thought best to put the patient under chloroform or ether. Let us first consider the methods of opening into the antrum from within the oral cavity. The first or second molar should be extracted. Then the floor of the antrum is to be cut out with chisels, gouges or drills, or with dental engine, the latter instruments probably being the ones more generally resorted to. There will not be much hemorrhage as no important vessels cross the operating field, though there may be considerable capillary oozing. Now, there is one other thing to be obtained here, viz., to get free drainage; do not hesitate to make an opening large enough, it should be excavated to admit the end of the little finger.

After sponging and cleaning away all debris and remains of the fluid enclosed within the sinus all those bony septi spoken of should be broken down, not leaving any pockets for foreign matter to accumulate. It will be necessary sometimes but rarely more often for the removal of solid growth to open the antrum from the outside of the face. When this is done the incision should be made through the soft tissues corresponding to a line about three-quarters of an inch above the gum margin opposite the second molar, and the outer antral wall be removed. This procedure will always leave a disfiguring scar as the muscle fibres have been cut nearly transversely and should not be used if at all avoidable. I can not imagine any cases in which this will be absolutely necessary, but I know that some of the best oral surgeons have thought they found this procedure unavoidable. After these steps in the operation have been completed the cavity should be thoroughly cleansed with a 1-2500 solution of bichloride of mercury. This will also do away with any bad odor if present; after it an injection of tincture of iodine will prove very efficient as astringent and stimulant. A piece of gum elastic catheter placed on the nozzel of the syringe will aid in this effectually and prevent the iodine from flowing over the surrounding soft tissues. Astringent disinfecting solutions should be used daily; a weak solution of permanganate of potash answers the purposes in many cases excellently, but if stimulation is required, as it is when the mucous membrane takes too long to recover a healthy condition, a weak solution of zinc chloride or sulphate may be used. This latter may be quite painful and a previous anodine injection may be indicated. Such solution may be a diluted tincture of opium to be injected and retained within for a few minutes; any other anodine may be just as well, but I am endeavoring to mention the drugs whose beneficial use I have seen proven. There has been another method in use viz.: tapping the antrum without the removal of any teeth by drilling through the mucous membrane where the cheek and bone meet, right above the second molar; but this plan will be found inefficient in many cases and will generally have to be followed by one of the others to obtain a cure. By opening into the sinus this way the most dependent point is not reached and this is the first thing desired for free drainage. Some have used still another method, and that was the introduction of a small silver tube. There are a number of objections to this, as the tube is frequently either too long or too short. If too long a certain amount of fluid will always remain in the bottom of the cavity. If it just reaches the floor of the antrum, or does not quite reach it, the lining mucous membrane will soon overlap the opening of the tube and fluids may be injected, but still not readily be evacuated. Should the discharge prove to be very watery, an astringent injection may be used, viz.: a diluted solution of hammamalis or nitrate of silver. If a large opening is obtained injections of peroxide of hydrogen will prove excellent. Saturated solutions of boracic acid are very good. During meals or sleep the artificial opening into the antrum should be plugged with a piece of disinfectant lint lest the accidental access of food may excite fresh irritation, or pus may be swallowed. Carbolized lint will be found better than cotton. Should the lint slip into the antrum it will be an easy matter to remove it by catching one end with a pair of tweezers, whilst should this happen to cotton, you will find it embarrassing and tedious to remove it from the cavity.

Whilst the abscess is imminent it may be sometimes prevented by removing a carious tooth in the immediate neighborhood, and the application of leeches will sometimes serve a good purpose. All antiphlogistic measures should be taken and the administration of purgatives not to be forgotten. Often alveolar abscesses open into the maxillary sinus but this is not to be regarded as a disease of the antrum.

Inflammation of the lining membrane of the antrum is of no different character from that of the nasal mucous membrane and is often concomitant with it. Also this trouble may originate through a diseased condition of the teeth. If the pulp of a tooth dies it decomposes in its own cavity and the putrefactive gases thereby produced pass through the apical foramen into the antrum if the roots penetrate its floor and create irritation and inflammation in the surrounding peridental membrane. The first symptoms of this disease are dull pain in that side of the face, and a dryness of the nostril on the affected side, generally accompanies the pain. The lining membrane of the antrum assists by its natural secretion of mucus in keeping the nostril moist. So too, when it participates with the nose in inflammation produced by a "cold in the head," it furnishes its mucoid and watery secretions which are so annoying in acute nasal catarrh. As everywhere in mucous membranes so also here, is the second symptom an increased flow of mucus. If only the nose were affected and not the antrum, the watery discharge would be of like amount from either nostril. In making a diagnosis this point will have to be taken into consideration. Should the opening from the antrum into the nose become closed the result would probably be a more purulent accumulation of fluid and there would be a considerable pain in the face, redness of the cheek, and more or less swelling of the soft tissue, due to the pressure within the cavity. The bone gradually becomes absorbed and the fistulous opening may point on the outside of the face, even on the lower jaw if not interfered with, which could be done, if timely noticed, by compressing and artificially opening the fistulous track into the mouth. In a healthy constitution a chronic inflammation of the antral mucous lining may continue for months without any general disturbances, or without exceeding its present boundaries, and it is often unappreciated, the patient supposing that he has but a nasal catarrh. Frequently the exudation drops into the posterior nares and then drips into the fauces instead of appearing in the nostril.

Treatment for this affection must be a general one. For an acute antral catarrh let the patient take a hot foot-bath. Give a night sweat; this can be accomplished by heating the feet with hot bricks or hot water in bottles and two or three tumblers of cold water taken internally on retiring. Also a six grain dose of sulphate of quinine may be given to relax the pores. Wrap the patient up well and be careful that no draft shall strike him whilst in this condition. A saline cathartic in the morning will prove very beneficial. Alteratives may be necessary to stimulate glandular action; when employed the doses should be small and continued for a greater length of time. The teeth should always be very carefully examined for the cause may be in one apparently sound. If a bad tooth be extracted in such a case it would probably be followed by a gush of purulent fluid. To obtain this effect it may be necessary to puncture the antrum through the alveolus, a thing very easily done and not at all accompanied with danger as there is no important structure that may be injured in the region. If medication is necessary be careful not to repeat it too often. Cauterizing agents are the ones mostly indicated. Inflammation of the lining membrane in a mild state may cause another disease termed dropsy or antracele, but if the inflammation goes on to a degenerating stage the result would be an abscess. It will sometimes be found quite difficult to differentiate between a chronic suppuration and solid growth, but acute inflammation is generally quite easy of diagnosis. An exploratory puncture is the best plan to do away with any doubt; such a puncture is not dangerous and may save the operator from a great blunder. Should the cavity be filled with liquid the patient may tell us of noticing a sensation of fluid washing about in the sinus whenever the head was suddenly moved.

Occasionally the root of a tooth may pass into the antrum during the attempt to extract it; such a mishap may occur in the hands of the most skillful operator. Sometimes the roots of upper molars penetrate the antral floor and enlarge within. If such a tooth be extracted the roots will either break and may slip into the sinus, or a portion of the floor will come away with it. Mr. Catlin relates where such a case in a young man had occurred. The father of this patient had died from malignant disease of the jaw; after consultation with several other surgeons it was thought best to remove the piece of root left behind in the antrum. The labial plate of the alveolus was punctured with a trephine and the cavity laid open. In this case the floor was divided into a number of pockets and this made the search for the fragment a long and tedious one; the artificial opening being properly treated healed very rapidly. If by falls or blows teeth should be driven into the antrum they should be promptly removed.

Antracele, or dropsy of the antrum is another affection of the mucous lining but does not differ very much from its inflammation. Some have claimed the first cause of the trouble to be a closing up of the antro-nasal opening but this remains to be proven; it may be that the closing of the opening is a symptom; or one of the results of the disease. Other causes lie in cystic degeneration of the mucous glands or inflammation of some part of the teeth, or any irritation causing a super-activity of the glands within the antrum. Antracele is also termed mucous engorgement and may be the result of a reflected chronic periodontial inflammation. The membrane at first becomes puffy and thickened and the secretions of the mucous glands become inspissated. The secretions of antracele Prof. Garretson says, from the mucous lining are similar to gonorrhœal discharge, indeed, an English writer, Mr. Bell, expresses the two conditions to be quite analogous. "Both disease," he says, "consisting equally in an altered secretion, in the one of the pituitary membrane, and in the other of the muscular lining of the urethra, which in neither instance possesses any of the characteristics of abscess, though the matter of both is purulent."

Do not think this comparison a very scientific one for as soon as the secretion in antrocele decomposes and thickens, forming pus, we have not antracele, but another disease termed abscess, and a comparison of fluids of this latter disease with that in gonorrhœa is more correct. The fluid is thin, looking somewhat like urine, or may be but slightly ropy. Puruloid secretions of the antrum may be of so limited a character, like the secretions of some ovarian cysts, that years may pass before marked inconvenience occurs. As a rule the diagnosis is not difficult. Generally, as already stated, it starts by a slight inflammation caused by a diseased tooth. The pain is sometimes sharp but at others unbearably dull, depending on the kind and rapidity of the accumulation within the cavity. The cheek becomes numb and heavy. The pain is about the same as already described under acute iuflammation of the mucous lining, only in the latter it is confined mostly to the alveolar arch, whilst in antracele it takes its seat in the orbital plate. As the fluid accumulates the parietes extend and the parts swell very much as in abscess of the antrum. Later on the mass may fluctuate.

Should the source of the trouble lay in some diseased tooth it should certainly be at once removed, but if some constitutional derangement be the cause, proper treatment should be applied and not as is often done blindly a molar extracted. The rule here again in treatment is first to *remove the cause* and next to attain *free drainage and cleanliness.* As you will readily see the further care is very much as that for abscess.

Cysts and cystomata are here to be considered. Cystic tumors is another name for cystomata. A cyst is a tumor, formed by a sac filled with fluid or a pulpy mass. If such a tumor develops from a sac already existing, it is called a cyst, but if it is developed entirely new, it is called a cystoma. Cystic sarcoma, cystic encrondroma are simply tumors, also connected with a cystic growth. The cyst in these cases forming the essential part of the tumor. The cause of cystic development in mucous membranes are inspissation of a gland, or closure of its secretory duct. If now, the secretion in a gland from either of the above causes be obtained, the secreting surface becomes expanded to a simple sphere; the enclosed secretions act as mechanical irritants and cause more or less inflammation of the surrounding parts, which consequently thicken, and surround the secretions like a sac. The closure of the secretory duct is usually the cause. The symptoms are nearly all local consisting in a general expansion of the jaw bone over the antral region, accompanied by a corresponding disfigurement of features. The source may lie in dental irritation, sometimes is caused by a misplaced tooth. Antral cysts have been known to reach the size of an ordinary orange. Diagnosis is very much like that of the afore-named troubles. Fluctuation can sometimes be produced, if the cyst is sufficiently large and if pressure or percussion to the antral wall be applied a parchment-like crackling sound will be noticed. Exploration here again will clear away any doubt in diagnosis. The treatment is first to open the cyst and to evacuate its fluid contents, then inject it with stimulating lotions; should the patient be one not very prone to inflammation, the cavity may be packed with absorbent cotton saturated with tincture of iodine. Several weeks are generally required for a complete cure. Let us now consider some of the solid growths in the antrum.

Tumors of the upper jaw are of somewhat the same nature as some in the lower jaw, but their effects are more serious and they cause greater deformity. The most common variety of tumors affecting the upper jaw, are sarcoma, carcinoma and osteoma. Enchondroma, fibroma and cystoma are comparatively rare. There are two forms of morbid growths found in the antrum, one consisting of hypertrophied glands, or glands undergoing cystic degeneration and the other allied to tubular epithelioma. The latter is a recurring and doubtless a malignant neoplasm. The signs and symptoms of tumors of the antrum are very much the same as those of the afore-named troubles; there is no increased discharge from the nose and no, or at least very little, escape of fluid after tapping. These tumors grow, commencing in the antrum, into the nose after expanding the wall of the sinus in all directions, and give rise to singular distortions of the features, obstruct the nasal passages and are often confounded with nasal polypi. They may occasion also absorption of the walls and protrude into the mouth and into the pharnynx or the base of the scull. To remove them,the anterior wall of the antrum will first have to be excised; when possible, this should be done through the mouth, without opening the face, by dissecting the soft parts away from the jaws and cutting the latter away.

Bony tumors of the jaw occur in two forms, growing either from the surface of the maxilla or from its cancellous portion.

The first may be regarded as an exostosis and have rarely anything to do with the antrum, unless their removal should require removal of part of the parietes. A frequent seat for osseous enlargement is seen in the tuberosity of the jaw, and may extend to the alveolus, including the floor of the antrum. Such a case was presented and successfully treated in a clinic at the Chicago College of Dental Surgery by Prof. T. W. Brophy last year. An interesting case is mentioned in Agnew's Surgery, one of Mr. Michous, an osseous tumor grew from the lower surface of the orbital plate of the frontal bone, filling the antrum. In another case the tumor had become detached from its bony seat of origin, and lay loose in the antrum. The structure of the osteomata of the jaw, either dense or ivory like, or soft and spongy. Those, commencing in the cancellous portion of the bone, are usually of the latter type; they seldom appear before middle age. The origin of tumors of the jaws is generally of an inflammatory sort. Diseased teeth, ill-fitting plates and sometimes blows upon the face are the most common causes. These tumors are usually of slow growth, are not very irregular on their surface, devoid of pain, and show no tendency to degenerate; they only become serious when their bulk obstructs the nasal passages, the mouth or orbit, or interferes with the movement of the jaws. As long as these tumors are small but little or no inconvenience, and no tendency to grow is present, no surgical interference is necessary. When limited to a portion of the jaw, or when occupying the antrum, the removal can be effected with chisels, gouges, bone-forceps, pliers, small saws or chainsaws. Should the disease have acquired the form of diffused hypertrophy, it will be necessary to extirpate the entire jaw. In all surgical operations on the superior maxillary, the orbital plate should be preserved, if at all possible.

Foreign bodies may occasionally find entrance into the antrum as bullets; they should be removed by opening the antral floor and the patient may be given proper antiphlogistic treatment. Diagnosis is not generally very difficult.

Many antral diseases are due principally to constitutional predisposing causes, such as scorbutus, mercurial stomatitis.

Scorbutic diathesis infers, mostly, to antral purulency and ulceration. The pathological changes in the maxillary sinus in scorbutic affections are similar to those of the mouth in general. Treatment for such cases have to be of constitutional kind, locally proper medications may be made with a fine trephine introduced above the canine fossa.

Scurvy and mercurial diathesis both act very similar by two different modes; they predispose through their constitutional relations and excite locally by inducing periodental inflammation. In the treatment, then, we will have to combat the effects of these two predisposing causes; first the poison must be eliminated from the system; chlorate of potash and the common muriate of soda are valuable medicines in this direction; next the pericementites is to be cured.

These are the most common affections of the maxillary sinus. There are many more and of difficult treatment and diagnosis. Great care should be taken in diagnosing these affections.

Authorities referred to: Garretson, Agnew, Holmes, Tomes, Hunter, Billroth, Heath.

---

TEMPERANCE INSTRUCTION IN PUBLIC SCHOOLS.—The report of the Department of Scientific Temperance Instruction in Public Schools for the year 1888 shows that twelve million children in this country are now under compulsory temperance education laws; that is to say, that the law has provided the education in favor of total abstinence that results from learning the nature and effects of alcoholic drinks and narcotics. This report further shows that there is no New England State without such a law; New Jersey is the only Middle State that has not enacted such a law; ten Southern and two Western States are still unprovided in that regard. The act of Congress of 1886 brought all the Territories under the law. Those interested in this subject will find reports from the different sections of the country of the work done, and the difficulties to be met and overcome in States in which as yet compulsory laws have not been enacted.

